# Efficacy of polymyxin B hemoperfusion in and beyond septic shock: is an “endotoxin severity score” needed?

**DOI:** 10.1186/s13054-018-2093-y

**Published:** 2018-08-17

**Authors:** Patrick M. Honore, David De Bels, Thierry Preseau, Sebastien Redant, Herbert D. Spapen

**Affiliations:** 1 ICU Department, Centre Hospitalier Universitaire Brugmann, Place Van Gehuchtenplein 4, 1020 Brussels, Belgium; 2 Emergency Department, Centre Hospitalier Universitaire Brugmann, Brussels, Belgium; 3 Universitair Ziekenhuis Brussel, VUB University, Brussels, Belgium

Polymyxin B (PMX) is a cationic polypeptide antibiotic which can bind and neutralize endotoxin. Parenteral administration of PMX, however, is associated with substantial nephrotoxicity limiting its clinical use. Immobilizing PMX to polystyrene-derived fibers in a cartridge integrated in a hemoperfusion device therefore represents an elegant method that permits adequate PMX therapy while avoiding systemic toxicity.

Nakamura et al. [1] recently reported that PMX hemoperfusion (PMX-HP) reduced intensive care unit-free days and all-cause hospital mortality in patients with septic shock caused by various pathogens and with various sites of infection. This large retrospective study adds support to PMX-HP as an adjunctive therapy of septic shock but remains vague and thought-provoking with regard to clarifying the reason(s) behind the observed beneficial effects of PMX-HP.

PMX-HP causes direct adsorption of circulating endotoxin and removes inflammatory cells and apoptotic factors [2]. Its ultimate effect reflects restriction of the endotoxic burden. Endotoxin is largely present in the outer membrane of Gram-negative (GN) bacteria. Hence, endotoxemia is typically associated with GN infection. However, it may also occur secondary to an impaired gastrointestinal (GI) barrier function. The GI tract is an important reservoir of “dormant” endotoxin. Any infectious (GN and Gram-positive (GP) bacteria, fungi, etc.) or noninfectious (multiple trauma, pancreatitis, hypovolemic shock, etc.) perturbation of GI permeability, in particular when associated with splanchnic hypoperfusion, may cause translocation and circulatory shedding of endotoxin [3]. Measuring endotoxin activity (EA) is thus a crucial incentive for selecting patients who may optimally benefit from PMX-HP. The Multicenter Endotoxin Detection in Critical Care study reported high (≥ 0.6) EA levels in 30% of patients with both “sterile” and infectious critical illnesses. An almost equal proportion of patients with GN and GP infection (6.9% vs 5.7%) had similar high EA levels. EA levels also correlated with sepsis severity [4]. EA levels were not measured in the PMX-HP trials investigating peritonitis-induced septic shock which may account for the divergent study results. The recently presented EUPHRATES study included patients with septic shock and an EA level ≥ 0.6. No difference in 28-day mortality was observed between PMX-HP-treated patients and controls except among subjects with a multiple organ dysfunction syndrome score above 9 and EA levels between 0.6 and 0.9 [5]. Taken together, effective PMX-HP treatment does not depend on the type of critical illness, microorganism, or infection site and might offer more patient-tailored benefit when based on measured EA levels.

## Abbreviations

EA: Endotoxin activity
GI: Gastrointestinal
GN: Gram-negative
GP: Gram-positive
PMX: Polymyxin B
PMX-HP: Polymyxin B hemoperfusion

## References

[1] Nakamura Y, Kitamura T, Kiyomi F, Hayakawa M, Hoshino K, Kawano Y, Yamasaki R, Nishida T, Mizunuma M, Ishikura H, Japan Septic Disseminated Intravascular Coagulation (JSEPTIC DIC) study group (2017). Crit Care.

[2] Ronco C, Klein DJ (2014). Polymyxin B hemoperfusion: a mechanistic perspective. Crit Care.

[3] De Jong PR, González-Navajas JM, Jansen NJG (2016). The digestive tract as the origin of systemic inflammation. Crit Care.

[4] Marshall JC, Foster D, Vincent JL, Cook DJ, Cohen J, Dellinger RP, Opal S, Abraham E, Brett SJ, Smith T (2004). Diagnostic and prognostic implications of endotoxemia in critical illness: results of the MEDIC study. J Infect Dis.

[5] Iba T, Fowler L (2017). Is polymyxin B-immobilized fiber column ineffective for septic shock? A discussion on the press release for EUPHRATES trial. J Intensive Care.

